# Nitrous Oxide Gas

**Published:** 1875-09

**Authors:** H. J. Barnes

**Affiliations:** Boston.


### SELECTED ARTICLES.

### ARTICLE III.

### *Nitrous Oxide Gas.*

BY H. J. BARNES, M. D., OF BOSTON.

Read before the Suffolk District Medical Society, October 31, 1874.

Since the discovery of the anæsthetic properties of ether, in 1846, the subject of anæsthesia has occupied so large a portion of our literature that, reluctantly, I invite your attention to another chapter.

But with the object of correcting what seem erroneous views respecting the administration of nitrous oxide gas, which have recently been published, and to show how it may be made useful and practicable, in place of ether, for painful examinations, short operations, and the adjustment of broken bones, this paper is prepared.

In the *Journal* of September 22, 1852, Vol. 48, No. 8, this subject was referred to in a communication by H. A. H., replying to the question, "Is nitrous oxide gas an anæsthetic?" The writer begs leave to reply, briefly, that it is not, and challenges the production of a case proving it is; after which, he cites the experiments of Davy, who said he breathed the gas for a week, and at the end had an increase of sensibility to pain, and it only proved to be intoxication. In a number of subsequent papers, in the same volume, these statements are refuted by numerous satisfactory experiments. But, since, that time, the subject seems to have attracted little attention, until a case of death from its use was reported in the *Lancet* of February, 1873, page 174 which, by the way, is the only disastrous result from its use

I have been able to find. The writer, in a review of the case, remarks "that a dentist stated to him 'that he never extracted a tooth until breathing had temporarily ceased;'" and then, in regard to this case, says "the first symptoms were rapidity of pulse, with diminution of volume, but the immediate cause of death was paralysis of respiration. Nitrous oxide gas, indeed, is not an anæsthetic at all. A true anæsthetic is an agent which suspends sensibility without, by any necessity, interfering with those processes on the continuation of which life depends. Nitrous oxide acts not in this way, but by suspending, for a brief period, one of the most important of the organic processes, that of respiration itself. For the reason named, the insensibility produced by the gas is a period of partial death. This interval, transient, doubtful, dangerous, may allow an operator time for a short operation, and, the agent away, the suspended function may return." A French chemist, writing recently on this subject, says "the gas only asphyxiates." Within a year, I have read an article in the Boston Medical and Surgical Journal, mentioning the attendant asphyxia when anæsthesia is produced by gas.

By the courtesy of the dental firm of Dr. Ball and Fitch, of this city, for the past four years, I have been permitted to use their office and gas for such operations as I saw fit, and I have had the advantage of many valuable suggestions as the result of their ten years' experience with the gas, during which time it has been administered by them not less than 15,000 times.

Ordinarily, a person is anæsthetized in from one to three minutes, by from twenty to one hundred inhalations of the gas, by which three or four gallons are consumed. The gentlemen spoken of recall but two patients who could not be brought under its influence. After breathing the gas continually for ten minutes, neither was sensibly affected in pulse, respiration or consciousness, and it was given up.

They remember a larger number, who, at the period of excitement, resisted its administration, and it was, for this reason, obliged to be suspended, they not having the means necessary to confine the patient, and compel the continuance of the inhalation through this stage.

Excitement, intoxication and narcotism are so distinctly observed as when ether is used, and this, too, without asphyxia, for this is accidental and not the necessary result.

The popular notion that it only asphyxiates, is perhaps, explained by the imperfect method of administration when first used, inhalation and exhalation being carried on through a single opening, into a rubber bag, holding from five to ten gallons, necessitating the continued breathing of air and gas used many times. This is now changed, and inspiration is from the gasometer; while expiration closes the opening, and is carried on through another communicating with the surrounding air.

Asphyxia is now a comparatively rare occurrence, particularly if we follow the rule I quote from Dr. Bigelow's paper, entitled, "Alleged Death from Ether," published last year in the *Journal* of Nov. 20th. "We should aim at anæsthesia by inebriation, not by asphyxia." If lividity be noticed when gas is used, a half turn of the stop-cock admits air, and the same relief is the result as when this condition follows the administration of ether and the sponge is removed.

The advantages in using gas in place of ether are briefly as follows:

The shorter time required to anæsthetize.

It is more agreeable.

There is rarely subsequent vomiting.

Its effects pass off quicker, three or four minutes being usually sufficient.

There is no danger of ignition when used.

The cost is very much less. Fifty dollars will purchase a complete apparatus for its manufacture, and about four cents a gallon will pay all other expenses.

It is made from the nitrate of ammonia, which is placed in a glass retort over a spirit lamp. It quickly melts, and, apparently boils, but it really undergoes decomposition, and is resolved into gaseous nitrous oxide and steam. It is passed through four jars, holding about two gallons each of water, and one, containing a solution of potash, to remove impurities. It is then conducted to the gasometer, and is ready for use.

The compressed or liquid gas is put in iron jars, holding what is equal to one hundred gallons of gas. These are filled, at six dollars each, and put in portable boxes, a foot and a half long, and about eight inches deep. With an inhaler, a rubber bag as a reservoir for the gas, and the necessary rubber tubing, the price is forty-eight dollars.

I have not observed hilarity as often in patients under its influence as with ether. A few pugnacious, excitable people are with difficulty carried through the different stages without a struggle. Usually, the gas is taken from this class when their ruling passion is manifested. After the excitement has passed, a little explanation of its effects dissipates their fear, and, with renewed inhalation, they go off quietly.

Ten minutes is the longest time I ever kept a patient under its influence. There were no unpleasant symptoms accompanying or following its use in this case, which was for the removal of a small tumor over the orbit. I should not hesitate to continue it longer, were it necessary, and, since writing this paper, I have been informed, by Dr. W. H. Baker, of its employment in two cases of ovariotomy, one of them lasting over an hour and a half, during his service in the Women's Hospital, of New York.

I have used it thirty-five or forty times in the removal of wens, opening abscesses and felons, the extraction of ingrowing nails, &c., and in each case the effect was satisfactory.

The expense and inconvenience of transportation will perhaps preclude its general employment in private practice, but, for hospitals, it seems particularly adapted.—*Boston Medical and Surgical Journal.*

